# A diagnostic PCR assay for the detection of an Australian epidemic strain of *Pseudomonas aeruginosa*

**DOI:** 10.1186/1476-0711-9-18

**Published:** 2010-07-16

**Authors:** Heidi L Williams, Lynne Turnbull, Susan J Thomas, Anna Murphy, Tim Stinear, David S Armstrong, Cynthia B Whitchurch

**Affiliations:** 1 Department of Microbiology, Monash University, VIC 3800, Australia; 2 The ithree Institute, University of Technology, Sydney, NSW 2007, Australia; 3 Department of Respiratory and Sleep Medicine, Monash Medical Centre, Clayton, VIC 3168, Australia; 4 Department of Paediatrics, Monash University, VIC 3800, Australia; 5 Department of Microbiology and Immunology, University of Melbourne, Parkville, VIC 3010, Australia

## Abstract

**Background:**

Chronic lung infection with the bacterium *Pseudomonas aeruginosa *is one of the hallmarks of cystic fibrosis (CF) and is associated with worsening lung function, increased hospitalisation and reduced life expectancy. A virulent clonal strain of *P. aeruginosa *(Australian epidemic strain I; AES-I) has been found to be widespread in CF patients in eastern Australia.

**Methods:**

Suppression subtractive hybridization (SSH) was employed to identify genetic sequences that are present in the AES-I strain but absent from the sequenced reference strain PAO1. We used PCR to evaluate the distribution of several of the AES-I loci amongst a collection of 188 *P. aeruginosa *isolates which was comprised of 35 AES-I isolates (as determined by PFGE), 78 non-AES-I CF isolates including other epidemic CF strains as well as 69 *P. aeruginosa *isolates from other clinical and environmental sources.

**Results:**

We have identified a unique AES-I genetic locus that is present in all 35 AES-I isolates tested and not present in any of the other 153 *P. aeruginosa *strains examined. We have used this unique AES-I locus to develop a diagnostic PCR and a real-time PCR assay to detect the presence of *P. aeruginosa *and AES-I in patient sputum samples.

**Conclusions:**

We have developed diagnostic PCR assays that are 100% sensitive and 100% specific for the *P. aeruginosa *strain AES-I. We have also shown that Whatman FTA^® ^Elute cards may be used with PCR-based assays to rapidly detect the presence of *P. aeruginosa *strains in CF sputum.

## Background

The emergence of "epidemic" strains of *P. aeruginosa *in cystic fibrosis (CF) clinics in Australia, the UK, Europe and Canada has been reported [1-8]. In Australia, the presence of a clonal isolate of *P. aeruginosa *was first detected following the death of five unrelated children attending the CF Clinic at the Royal Children's Hospital, Melbourne [9,10]. Ensuing surveillance of the CF clinic in 1999 found that 55% of those infected with *P. aeruginosa *had an identical or closely related strain to that of the five young children [10]. This strain also appeared to show increased virulence as it was associated with increased hospitalisation and poorer pulmonary function amongst infected patients [10,11]. We also reported a case of cross-infection of a patient with non-CF bronchiectasis that resulted in significant clinical deterioration [12]. This strain has also been identified in CF clinics in Sydney and Brisbane [1] and was previously referred to as m16 [10], pulsotype I [1] or C3789 [13]. To avoid further confusion we now refer to this strain as the Australian epidemic strain-I (AES-I).

Two hospital environmental studies in 1995 and 1999 failed to identify a common source for AES-I, suggesting person-to-person transmission or cross-infection [10]. To halt further dissemination of this strain, we elected to institute strict cohort segregation measures whereby CF children with AES-I infection were physically separated from other CF children in the hospital wards and outpatient clinics. Three years after the introduction of these measures, we noted a significant decrease in the incidence and prevalence of AES-I infection [14]. This observation strongly supports the notion that the AES-I is transmitted by cross-infection between patients during hospital and clinic visits and highlights the success of cohorting strategies in limiting AES-I transmission.

AES-I strains isolated from CF patient sputa are currently identified via pulsed field gel electrophoresis (PFGE). PFGE is an expensive, time consuming technique requiring highly skilled personnel and thus cannot be used as a means of routine surveillance for the presence of the AES-I strain in patients attending CF clinics. There is an urgent need for the development of a simple, rapid diagnostic method that will enable routine surveillance for AES-I in Australian CF clinics so that appropriate segregation measures can be instituted in an expeditious manner.

The development of diagnostic PCR-based assays have proven to be effective tools for the rapid detection of pathogens in infectious diseases, including other known clonal *P. aeruginosa *strains [13,15-20]. Our aim in this study was to determine if there were genetic sequences that are unique to the AES-I that might be used to develop a diagnostic PCR assay to enable rapid detection of this strain directly from CF patient sputum.

## Methods

### Bacterial strains

*Pseudomonas aeruginosa *strains used in this study are listed in Table 1. *Eschericia coli *strain DH5α was used for transformation of the suppression subtractive hybridization (SSH) library.

**Table 1:** *Pseudomonas aeruginosa *strains used in this study

Strain type	Number or identity	Source	PFGE typed prior to this study
Laboratory	PAO1 (ATCC 15693), PAK, PA103, ATTC 27853		
CF isolates	56 (sputum, bronchial lavage)	Royal Children's Hospital, Melbourne	Yes [10]
CF isolates	54 (sputum)	Monash Medical Centre, Melbourne	No
Non-CF isolates	7 (urine, sputum, bronchial lavage)	Monash Medical Centre	No
Non-CF isolates	52	Gribbles Pathology, Melbourne	No
Environmental	6	Water Board Authority, Sydney	No
Known clonal/epidemic	Liverpool (LES431, LESB58), Manchester (8799, C3373), Midlands (8916, 10066), Stoke and Trent	Craig Winstanley (University of Liverpool, Liverpool, UK)	Yes [2,8,27]
Known clonal/epidemic	Australian Epidemic Strain II (AES-II)	Claire Wainwright (Royal Children's Hospital, Brisbane)	Yes [27]

### Molecular techniques

Chromosomal DNA for SSH was isolated using the Masterpure DNA Purification Kit (Epicentre Technologies). Chromosomal DNA used for PCR was isolated using Masterpure DNA Purification Kit, DNeasy Tissue Purification Kit (Qiagen Sciences) or Whatman FTA^® ^Elute cards. Miniprep DNA of SSH clones was prepared using the QIAprep Spin Miniprep Kit (Qiagen Sciences).

### Construction and screening of AES-I subtraction library

The AES-I isolate (strain 973) was used as the tester strain for SSH. This strain was chosen from a collection obtained from the Royal Children's Hospital, Melbourne and had been previously identified as the clonal strain AES-I [10]. The driver strain used for SSH was the sequenced reference strain PAO1. The Clontech PCR-Select Bacterial Genome Subtraction kit was used to generate the SSH library of *Rsa*I fragments according to manufacturer's instructions but with a hybridization temperature of 73°C. The library of SSH PCR amplicons was cloned in pGEM-T Easy (Promega). Individual clones were miniprepped and sequenced. Nucleotide sequences were determined by use of a PRISM Big Dye Terminator Cycle Sequencing Ready Reaction kit (Applied Biosystems) according to the manufacturer's instructions and separated by capillary electrophoresis on an Applied Biosystems 3730S Genetic Analyser at the Monash University Micromon sequencing facility. Sequence data was analysed using MacVector (Accelrys). To determine the presence or absence of the SSH clone sequence in the PAO1 genome, sequences were used in BLASTN searches at the *Pseudomonas *genome project web-site [21]. AES-I sequences were further analysed using BLASTN and BLASTX searches [22] of the NCBI Genbank databases http://www.ncbi.nlm.nih.gov.

### PCR screening of strains

Oligonucleotides (Sigma Proligo) and annealing temperatures used in the PCR assays are listed in Additional File 1. Primers to amplify AES-I genome sequences were designed using MacVector (Accelrys). Primers to amplify the conserved *oprL *gene have been described previously [23]. Amplifications were carried out with an initial denaturation of 94°C for 2 min followed by 40 cycles consisting of 94°C (1 min), annealing temperature (1 min) and 72°C (1 min) with an additional extension at 72°C for 10 min following completion of the 40 cycles. Reaction products were electrophoresed on 2% (wt/vol) agarose gels to determine the presence of the appropriately sized amplicon.

### Pulsed Field Gel Electrophoresis

Molecular typing by PFGE following digestion with *Spe*I and *Dra*I was performed as previously described [24]. An isolate was considered to be AES-I or closely related to AES-I if its PFGE patterns differed by no more than three bands from the *P. aeruginosa *strain 973 [10].

### PCR from sputum samples

Samples of sputum were swabbed onto Whatman FTA^® ^Elute cards and allowed to air dry. DNA from a 2 mm diameter disc from each card was eluted into 50 μL of water as per the manufacturer's instructions and added to PCR tubes containing reaction mixtures for the PCR amplification of either *oprL *or the SSH-identified AES-1 genomic locus HW2. *P. aeruginosa *strains were also isolated from the sputum samples and typed by PFGE to validate the PCR diagnosis.

### Quantitative Real-time PCR

Primers and TaqMan^® ^MGB probes (Applied Biosystems) were designed from regions of the sequences of the HW2 locus and the *oprL *gene using the Primer Express^® ^Software v1.0 program (Applied Biosystems). Probe RTHW2-P (Additional File 1) was labelled with fluorescent dye 6-FAM at the 5' end and nonfluorescent quencher BHQ1 at the 3' end (Sigma). Probe RT*oprL*-P (Additional File 1) was labelled with fluorescent dye 6-TAMRA at the 5' end and nonfluorescent quencher BHQ2 at the 3' end (Sigma). Real-time PCR mixtures contained, 0.8 μM concentrations of each primer, 0.6 μM concentration of the probe, ABsoluteTM QPCR ROX (500 nM) Mix (ABgene) and either 1 μl (genomic) or 5 μl (Whatman FTA^® ^Elute card) of template DNA. To monitor PCR inhibition the TaqMan^® ^Exogenous Internal Positive Control (IPC) (Applied Biosystems) was multiplexed in the HW2 assay. The *oprL *assay was performed as a singleplex reaction. Amplification and detection were performed with the Eppendorf Realplex Mastercycler using the following program: 1 cycle of 95°C for 15 min and 40 cycles of 95°C for 15 sec and 60°C for 1 min. Negative controls were included in each assay. PCR products were also visualized by agarose gel electrophoresis to verify product amplification of the expected size. Cycle thresholds (Ct) from experiments to determine method sensitivity were reported as the average and standard deviation of three biological repeats. Correlation coefficients between Ct and DNA template concentration for each TaqMan assay were calculated by regression analysis and amplification efficiency was calculated using the formula: efficiency = -1 + 10^(-1/slope). Primers and probes for quantitative real-time PCR for AES-I are available as a kit through Cardinal Bioresearch http://www.cardinalbioresearch.com.au.

## Results

### AES-I subtracted Library

To identify genes unique to the AES-I genome, we performed suppression subtractive hybridisation (SSH) of DNA from AES-I isolate 973 against PAO1, the sequenced reference strain of *P. aeruginosa*. We sequenced 20 clones from the SSH clone library and identified 14 that contained AES-I sequences not present in PAO1 (Additional File 2) and of these 3 (HW1, HW11 and HW20) encoded identical sequences. BLASTX analysis of the 12 unique AES-I SSH sequences indicated that 3 (HW15, HW16, HW21) were 100% identical to genes located within the *P. aeruginosa *O6 antigen LPS biosynthetic gene cluster [25] and thus identified AES-I as belonging to the O6 serotype, a widespread serotype and the same as the LES and Midlands epidemic strains [13]. Other homologies include a probable bacteriophage tail protein (HW8), a very short mis-match repair protein (HW6), a DNA cytosine methyltransferase (HW6), a restriction endonuclease (HW12) and a dGTP triphosphohydrolase (HW22) (Additional File 2). Interestingly loci HW1/11/20, HW18 and HW23 encode putative proteins with 100% identity to 2 hypothetical proteins of the sequenced Liverpool epidemic strain LESB58 (Additional File 2). BLASTN and BLASTX searches with the remaining 2 clones (HW2 and HW3) did not produce significant hits in the Genbank sequence databases (Additional File 2).

### Distribution of AES-I sequences in *P. aeruginosa *isolates

We designed PCR primer pairs to amplify each of the 9 different AES-I genetic sequences that were not found in PAO1 and that did not localise to the O6 antigen gene cluster. We first performed PCRs with each primer set using the AES-I SSH driver strain (isolate 973) and PAO1 genomic DNA to confirm that the PCR reaction worked and was specific to AES-I. One of the primers sets (HW1/11/20) amplified a non-specific product in PAO1 and was not used in further assays.

We next performed an initial PCR profiling with the remaining 8 AES-I PCR primer sets on a collection of 9 AES-I strains (including driver strain), 9 non-AES-I CF isolates, 7 non-CF clinical isolates, 6 environmental isolates and the common laboratory strains PAO1, PA103, PAK and ATCC 27853 (Table 2). Each of the CF isolates in this collection had been previously genotyped by PFGE and classified as AES-I or non-AES-I [10]. We also performed PCR with primers that were designed to amplify the conserved *oprL *gene that is present in all *P. aeruginosa *strains [23,26] to confirm that a negative PCR result was not due to failure of the PCR reaction. These PCR assays showed that only the SSH loci HW2 and HW3 were unique to all 9 AES-I strains tested (Table 2).

**Table 2:** Distribution of AES-I genetic loci identified by suppression subtractive hybridisation in *P. aeruginosa *strains

Isolates	*oprL*	HW2	HW3	HW6	HW8	HW12	HW18	HW22	HW23
**Initial screen**									
AES-I (n = 9)	9	9	9	9	9	9	9	9	9
non AES-I CF (n = 9)	9	0	0	1	0	0	0	1	2
non-CF clinical (n = 7)	7	0	0	1	1	1	5	0	2
Lab strains (n = 4)^a^	4	0	0	0	0	0	2	1	2
Environmental (n = 6)	6	0	0	0	1	0	2	0	0

^a ^PAO1, PAK, PA103 and ATCC 27853

The *oprL*, HW2 and HW3 PCR primer sets were then used to screen a larger collection of *P. aeruginosa *strains including a further 38 CF isolates that had been previously profiled by PFGE typing to be either AES-I (18) or non-AES-I (20) [1,10], 9 representatives of the Liverpool, Manchester, Midlands, Stoke, Trent and Australian pulsotype II epidemic strains [2,8,27] as well as another 52 non-CF clinical isolates (Table 3). These assays revealed that the HW2 locus was present in all of the additional 18 AES-I isolates and none of the other *P. aeruginosa *strains tested whereas HW3 was found to be present in all but 4 AES-I strains and none of the other *P. aeruginosa *strains (Table 3). We also performed BLASTN analyses of the genome sequences of the *P. aeruginosa *strains PA14, PA7, PACS2, PA2192, and PAC3719 to determine the presence of the HW2 and HW3 loci in these genomes and found that neither locus is present in any of these sequenced strains.

**Table 3:** Further screening of the distribution of AES-I genetic loci in *P. aeruginosa *strains

Isolates	*oprL*	HW2	HW3
**Second screen**			
AES-I (n = 18)	18	18	14
non AES-I CF (n = 29)^a^	29	0	0
non-CF clinical (n = 52)	52	0	0
**CF clinic screen**			
AES-I (n = 8)	8	8	7
non AES-I CF (n = 46)	46	0	0

^a ^includes Liverpool, Manchester, Midlands, Trent, Stoke and AES-II epidemic strains

### Detection of AES-I in *P. aeruginosa *isolates from a CF clinic

In 2005 we commenced a study to determine the prevalence of the AES-I strain in patients attending the Monash Medical Centre CF clinic. Sputum was collected by spontaneous expectoration or hypertonic saline induction from patients 6 years of age or older and plated onto selective media to detect *P. aeruginosa *by standard techniques [28]. PFGE was used to identify the genotype of 54 *P. aeruginosa *isolates of which 8 were identified to be AES-I. PCR with the *oprL*, HW2 and HW3 primer sets was performed on DNA purified from the same set of *P. aeruginosa *isolates (Table 3). These PCR assays showed that the HW2 and HW3 loci were absent in all 46 non-AES-I *P. aeruginosa *isolates obtained in this study. Each of the 8 AES-I strains possessed the HW2 locus whereas the HW3 locus was present in 7 of the 8 AES-I isolates (Table 3).

### PCR detection of *P. aeruginosa *and AES-I in CF sputum using Whatman FTA^® ^Elute cards

**Figure 1:**
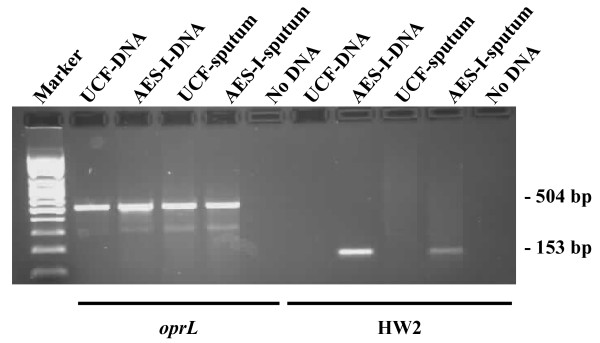
**PCR detection of *oprL *and the AES-I HW2 locus from CF sputum**. Sputum from 2 patients that had been shown by PFGE screening to contain either AES-I or a unique strain of *P. aeruginosa *(UCF) was swabbed onto Whatman FTA^® ^Elute cards. The *oprL *and HW2 loci were amplified by PCR from these sputum samples (UCF-FTA, AES-I-FTA) or chromosomal DNA (UCF-DNA, AES-I-DNA) purified from the *P. aeruginosa *strains isolated from the same sputum sample. These strains were also confirmed by PFGE to be AES-I or a unique strain of *P. aeruginosa *(data not shown).

We explored the potential use of Whatman FTA^® ^Elute cards to purify DNA for use in PCR assays for the rapid detection of *P. aeruginosa *strains directly from patient sputum. A related technology, the FTA^® ^card (Whatman), has been shown to be an efficient method to store, transport and isolate DNA from sputum samples suspected to harbor *Mycobacterium tuberculosis *[29]. The FTA^® ^Elute technology has further simplified the elution of DNA for PCR and is readily adaptable for routine pathology testing. We used PCR assays for the *oprL *and HW2 loci to determine if we could detect the presence of *P. aeruginosa *and AES-I directly from sputum samples stored on Whatman FTA^® ^Elute cards. Sputum samples from Monash Medical Centre CF clinic patients that were positive or negative for the AES-I strain as determined by PFGE, were swabbed onto Whatman FTA^® ^Elute cards, DNA eluted and used in the *oprL *PCR assay to determine the presence of *P. aeruginosa *in the sputum sample and in a 2^nd ^PCR assay with the HW2 AES-I primers to determine if the *P. aeruginosa *present in the sputum was AES-I (Fig 1). The results of these PCR assays showed that whilst both sputum samples were positive for the presence of *P. aeruginosa *as determined by the *oprL *PCR, only the sputum from the patient harbouring AES-I was positive with the HW2 PCR assay. These results demonstrate the potential utility of using Whatman FTA^® ^Elute cards in PCR-based assays to rapidly determine the presence of *P. aeruginosa *and AES-I directly from sputum.

### Development of a real-time PCR protocol to detect AES-I

To improve the diagnostic utility of the HW2 PCR we then developed real-time PCR TaqMan assays for *oprL *and the HW2 locus. Detection sensitivity of the HW2/IPC multiplex and *oprL *TaqMan assays was assessed by performing real-time PCR on dilutions of purified *P. aeruginosa *genomic DNA from an AES-I strain. The standard curves obtained with 10-fold serially diluted genomic DNA preparations were linear over seven orders of magnitude for HW2 and five orders for *oprL *(Table 4). Based on the complete DNA sequence, the predicted mass of a single copy of the *P. aeruginosa *PAO1 6,264,404 bp genome is 4.5 fg. Assuming a single copy of the target in the *P. aeruginosa *AES-I genome, the HW2 assay detected at least 2.2 × 10^2 ^genome copies. The *oprL *assay was less sensitive, with a detection limit of 2.2 × 10^4 ^genomes. The HW2/IPC and *oprL *assays were also evaluated as a triplex reaction but detection sensitivities were reduced (data not shown).

**Table 4:** Sensitivity of TaqMan PCR for detection of *oprL *and the AES-I HW2 locus

DNA per reaction (ng)	**Genome equivalents/reation**^**a**^	*oprL*		HW2	
		**Average Ct TAMRA**^**b,d**^	Standard Deviation	**Average Ct FAM**^**c,d**^	Standard Deviation
1000	2.2 × 10^8^	25.44	1.99	16.30	0.47
100	2.2 × 10^7^	29.13	3.19	19.57	0.27
10	2.2 × 10^6^	32.77	2.86	23.00	0.68
1	2.2 × 10^5^	35.18	2.23	26.40	0.95
0.1	2.2 × 10^4^	37.47	1.04	30.19	0.90
0.01	2.2 × 10^3^	-	-	33.23	0.84
0.001	2.2 × 10^2^	-	-	36.70	1.46

^a ^based on weight of *P. aeruginosa *PAO1 genome of 4.5 fg

^b ^*oprL *correlation coefficient r^2 ^= 0.9865, amplification efficiency = 114.84%

^c ^HW2 correlation coefficient r^2 ^= 0.9996, amplification efficiency = 96.84%

^d ^Fluorescent dye used for primer labeling; see Materials and Methods.

Next, the specificity of the two TaqMan assays was tested against purified DNA from *P. aeruginosa *PAO1, *P. putida and P. fluorescens*. The HW2 TaqMan assay was negative for these samples while *oprL *was positive for both *P. aeruginosa *and *P. putida*. DNA was then extracted from Whatman FTA^® ^Elute cards spiked with AES-I positive and unique strains of *P. aeruginosa*. As predicted, all extracts were *oprL *positive and only extracts from *P. aeruginosa *AES-I strains were HW2 and *oprL *positive. The same pattern of results was obtained with DNA extracted from the AES-I positive and negative sputum samples stored on Whatman FTA^® ^Elute cards used in the conventional PCR assay (Fig 1, Table 5). These data suggest that the HW2 and *oprL *TaqMan assays may have sufficient sensitivity and specificity for screening clinical samples using sputum collected on Whatman FTA^® ^Elute cards.

**Table 5:** Specificity of TaqMan PCR for detection of *oprL *and the AES-I HW2 locus

		TaqMan assay	
**Sample**^**a**^	No of samples tested	*oprL*	HW2
		**Ct TAMRA**^**b**^	**Ct FAM**^**b**^
AES-I sputum	1	31.61	21.33
Non-AES-I sputum	1	28.45	-
AES-I strains	31	23 - 30	18 - 30
Non-AES-I strains	8	28 - 30	-

^a ^All samples were purified using Whatman FTA Elute cards

^b ^Fluorescent dye used for primer labeling; see Materials and Methods.

## Discussion

AES-I is a highly transmissible virulent strain of *P. aeruginosa *that has been identified in several CF clinics along the eastern seaboard of Australia where surveillance programs have been introduced [1,10]. We have previously shown that patient segregation has successfully limited the spread of AES-I in a CF clinic in Melbourne [14]. There is currently an urgent need for a diagnostic tool that will enable rapid identification of CF individuals that harbour the AES-I strain so that appropriate segregation measures can be employed during hospital and clinic visits. Toward this end, we have used SSH and PCR to identify 2 genetic loci (HW2 and HW3) that are highly conserved amongst AES-I isolates of *P. aeruginosa*. PCR was utilised to profile the presence of these AES-I loci in a total of 188 *P. aeruginosa *strains. These screens found that the HW3 locus was absent in all 153 non-AES-I isolates but present in only 30/35 of the AES-I strains (86% sensitive and 100% specific) indicating that this locus is present in a region of some variability between AES-I isolates. The HW2 locus was found to be present in all 35 strains determined by PFGE to be AES-I and absent in all 153 non AES-I *P. aeruginosa *strains tested. These results indicate that PCR for the HW2 locus is 100% specific and 100% sensitive for detection of the AES-I epidemic *P. aeruginosa *strain.

The collection of strains used in this study included isolates from CF individuals that were identified in our initial 1999 clinic surveillance to harbour the AES-I strain [9,10] as well as isolates obtained over the subsequent 6 years from the same CF individuals. The HW2 locus was present in all initial and subsequent AES-I isolates obtained from the same individual indicating that this locus did not display variability during chronic infection over this time period.

The main purpose of the *oprL *PCR assay in our study was to act as a positive control for DNA quality to avoid false negative results in our PCR screens. Since we commenced our clinical surveillance study in 2005, it has been shown that the *ecfX *locus is more specific than *oprL *to identify *P. aeruginosa *from various environmental and clinical extracts [30]. Accordingly, a PCR assay for the *ecfX *locus could be substituted for the *oprL *assay to provide greater specificity for the presence of *P. aeruginosa *in sputum samples if desired.

Compared with PFGE which can take 6 to 8 days to obtain a diagnosis from time of receipt of patient sputum, PCR-based assays have many benefits as potential diagnostic tools as they are simple, relatively inexpensive, do not require highly skilled personnel, reduce handling and detection errors and can significantly reduce the time required to identify patients harbouring the AES-I strain. We have shown that PCR amplification of the *oprL *and HW2 loci can be used for the direct detection of *P. aeruginosa *and AES-I in CF sputum swabbed onto Whatman FTA^® ^Elute cards. We are now further assessing the potential for these assays for use as routine surveillance tools for *P. aeruginosa *and AES-I in CF sputum. The use of Whatman FTA^® ^Elute cards for sputum storage, transport and DNA template purification for PCR is simple and relatively inexpensive and could be easily adapted for detection of other bacterial pathogens from a broad range of respiratory and other infections.

## Competing interests

Monash University has a patent application pending for the AES-I sequences identified in this study. CBW will receive a percentage of royalties arising from commercialisation of the AES-I diagnostic kit according to Monash University policy. The other authors of this manuscript declare no personal, professional or financial relationships that cause a conflict of interest that could bias this work.

## Authors' contributions

HLW carried out the subtractive hybridization study, designed the PCR screen and performed the initial screening of lab and clinical isolates. LT participated in the design of the study, carried out screening of the clinical isolates, analysed the data and helped to draft the manuscript. SJT screened additional clinical isolates and carried out the real-time PCR conversion of the assay and screened clinical isolates. AM co-ordinated collection of samples from patients and helped with data analysis and interpretation. TS participated in design and co-ordination of the real-time PCR assay conversion and helped draft the manuscript. DSA participated in the concept and design of the study and data interpretation. CBW conceived, designed and co-ordinated the study and drafted the manuscript. All authors have read and approved the final manuscript.

## Supplementary Material

Additional file 1 **PCR Primer sequences used in this study**. Click here for file

Additional file 2 **Homologies of AES-I genomic sequences identified by suppression subtractive hybridization (SSH)**. Click here for file
